# The Effect of Endurance and Endurance-Strength Training on Bone Mineral Density and Content in Abdominally Obese Postmenopausal Women: A Randomized Trial

**DOI:** 10.3390/healthcare9081074

**Published:** 2021-08-20

**Authors:** Małgorzata Jamka, Edyta Mądry, Paweł Bogdański, Jakub Kryściak, Radosław Mądry, Aleksandra Lisowska, Elnara Ismagulova, Anna Gotz-Więckowska, Izabela Chudzicka-Strugała, Ainur Amanzholkyzy, Jarosław Walkowiak

**Affiliations:** 1 Department of Pediatric Gastroenterology and Metabolic Diseases, Poznan University of Medical Sciences, Szpitalna Str. 27/33, 60-572 Poznań, Poland; mjamka@ump.edu.pl; 2 Department of Physiology, Poznan University of Medical Sciences, Święcickiego Str. 6, 60-781 Poznań, Poland; emadry@ump.edu.pl; 3 Department of Treatment of Obesity, Metabolic Disorders and Clinical Dietetics, Poznan University of Medical Sciences, Szamarzewskiego Str. 82, 60-569 Poznań, Poland; pbogdanski@ump.edu.pl; 4 Department of Physiology and Biochemistry, Poznan University of Physical Education, Królowej Jadwigi Str. 27/39, 61-871 Poznań, Poland; j.krysciak@awf.poznan.pl; 5 Department of Oncology, Poznan University of Medical Sciences, Szamarzewskiego Str. 84, 60-569 Poznań, Poland; rmadry@ump.edu.pl; 6 Department of Clinical Auxology and Pediatric Nursing, Poznan University of Medical Sciences, Szpitalna Str. 27/33, 60-572 Poznań, Poland; alisowska@ump.edu.pl; 7 ENT Diseases Department, West Kazakhstan Marat Ospanov State Medical University, Maresyev Str. 68, Aktobe 030019, Kazakhstan; ismagulova_e@mail.ru; 8 Department of Ophthalmology, Poznan University of Medical Sciences, Szamarzewskiego Str. 84, 60-569 Poznań, Poland; agotzwieckowska@ump.edu.pl; 9 Department of Medical Microbiology, Poznan University of Medical Sciences, Wieniawskiego Str. 3, 61-712 Poznań, Poland; izachudzicka@ump.edu.pl; 10 Department of Normal Physiology, West Kazakhstan Marat Ospanov State Medical University, Maresyev Str. 68, Aktobe 030019, Kazakhstan; a.ainur.82@mail.ru

**Keywords:** bone, densitometry, exercise, obesity, postmenopause

## Abstract

The optimal type of exercise that simultaneously decreases body weight and preserves bone health in people with obesity is unknown. This parallel randomized trial aimed to compare the effect of endurance and endurance-strength training on bone mineral density (BMD) and content (BMC) in abdominally obese postmenopausal women. A total of 101 women were recruited and randomly assigned to endurance or endurance-strength training groups. Participants trained for 60 min per day, three times per week for 12 weeks. The endurance exercises were performed at an intensity of 50–75% of the maximum heart rate, whereas the strength exercises were at 50–60% of the one-repetition maximum. Pre- and post-intervention BMD and BMC of the total body, lumbar spine, and femoral neck and physical capacity were measured. There were no differences among the densitometric parameters in the endurance group, but a significant increase in whole-body BMD in the endurance-strength group was found. Moreover, there was a significant difference between the groups in the changes in the lumbar spine BMC. Furthermore, both training programs significantly improved physical capacity with no differences between groups. Endurance training was more effective in maintaining BMC at the lumbar spine. However, both groups did not differ in effect on BMD. Further studies with a long-term follow-up should be considered to confirm these findings. The study was registered with the German Clinical Trials Register within the number DRKS00019832, and the date of registration was 26 February 2020 (retrospective registration).

## 1. Introduction

Several studies have shown that obesity is related to a high bone mass [1] while decreases in body weight might cause bone loss [2]. Indeed, it has been reported that a higher body weight is associated with a heavier mechanical loading of bones, therefore enhancing the differentiation of osteoblasts. Moreover, weight gain increases the number of adipocytes, which are essential estrogen sources, resulting in increased bone mineral density (BMD) [3]. It should be highlighted that, in women, estrogen levels are an important determinant of bone health and that the relationship between estrogen depletion and bone loss is well established [4]. Insulin resistance can also increase circulating quantities of sex hormones, thereby increasing bone mass [5]. However, it has been also suggested that a high body weight might negatively affect bone health [6]. The risk of osteoporosis, osteopenia, or non-spine fractures increases in subjects with a higher percentage of body fat, probably due to the replacement of osteoblasts in bone by adipocytes [7]. Furthermore, adipokines levels might play important roles in bone formation and resorption [8].

Physical activity is one of the most effective non-pharmacological methods for improving or maintaining BMD and bone mineral content (BMC) [9]. Physical training can also attenuate the decrease in BMD, which is frequently observed during weight loss [2,10]. Exercises may directly or indirectly act on bone cells, affecting many aspects of bone remodeling [11] as well as promoting the release of growth hormone, prostaglandins, parathyroid, and thyroid hormones, which play important roles in bone formation [12,13]. The mechanical loading of bones during training depends on the type, intensity, and duration of exercise. Moreover, the effect of a training program on bone mass might vary according to age, sex, and site of measurements [9] and may differ in pre- and postmenopausal women [14,15].

Nevertheless, exercises generating high-intensity loading are more effective in increasing or maintaining BMD [16]. However, the type of exercise that simultaneously decreases body weight and maintains bone health in people with obesity is still unknown. The World Health Organization guidelines highlight the importance of regularly undertaking both endurance and resistance exercises [17], while the American College of Sports Medicine recommended moderate-to-high intensity aerobic or weight-bearing exercises to maintain or ascend bone mass [18]. Indeed, several studies indicated that endurance [19], strength [20], and combined [21] training are generally associated with an increase or maintenance of BMD or BMC. In contrast, Beavers et al. [22] suggested that strength training rather than endurance training had a more osteogenic effect. However, little is known about whether endurance-strength training is also more effective on bone health than endurance training alone. Currently, only a few studies have compared the effects of endurance and endurance-strength training on densitometric parameters in obese subjects and provided conflicting results [21,23,24]. Moreover, most of the studies were conducted on the adolescent population [21] or included dietary intervention together with training [23].

Therefore, this study aimed to compare the effect of endurance and endurance-strength training on BMD and BMC in abdominally obese postmenopausal women. Moreover, to clarify the effectiveness of the training programs, physical capacity was assessed.

## 2. Materials and Methods

### 2.1. Study Overview

This study was designed as a prospective parallel randomized trial and approved by the Poznan University of Medical Sciences Bioethical Committee (refs. 219/16 ad 1155/18). The study was conducted according to the guidelines in the Declaration of Helsinki. The study protocol was registered (retrospective registration) in the German Clinical Trials Register database (https://www.drks.de/; accessed on 16 November 2020) with the registration number: DRKS00019832 (date of registration: 26 February 2020). The study was per the standards of CONSORT (see Supplementary Material, Table S1) [25]. The full study protocol was described in detail previously [26].

### 2.2. Participants

The subjects were recruited from the Department of Treatment of Obesity, Metabolic Disorders, and Clinical Dietetics, Poznan University of Medical Sciences, as well as by promoting the project at the university among office, laboratory, teaching, and technical employees. All candidates underwent medical screening and interviews in the Department of Treatment of Obesity, Metabolic Disorders, and Clinical Dietetics. Postmenopausal women were eligible for inclusion if they were 50–60 years old, had abdominal obesity (defined as body mass index (BMI) ≥30 kg/m^2^, waist circumference >80 cm, and body fat ≥32% (recommendation of the American Council on Exercise [27])), and stable body weight in one month before the study. The exclusion criteria included previously diagnosed type 2 diabetes mellitus, cancer, secondary obesity or hypertension; poorly controlled blood pressure (systolic blood pressure >140 mmHg and diastolic blood pressure >90 mmHg) during the one month before the study; modification of their hypertension or lipid abnormality treatment, which required pharmacological treatment in the last three months prior to the trial; previously diagnosed coronary heart disease, heart failure, stroke, arrhythmias, and other conduction disorders; liver, kidney, or thyroid diseases; acute or chronic inflammation within the respiratory, gastrointestinal, or genitourinary systems; infection in the last month before the study; arthritis; connective tissue disease; tobacco, alcohol, or drug addiction; pregnancy, lactation, or childbirth within three months before the study; any other diseases that may prevent the subjects from participating or cooperating in the protocol; and the use of any dietary supplements during the last three months before enrolment. All participants provided written informed consent and were examined by a physician.

### 2.3. Intervention

**Figure 1:**
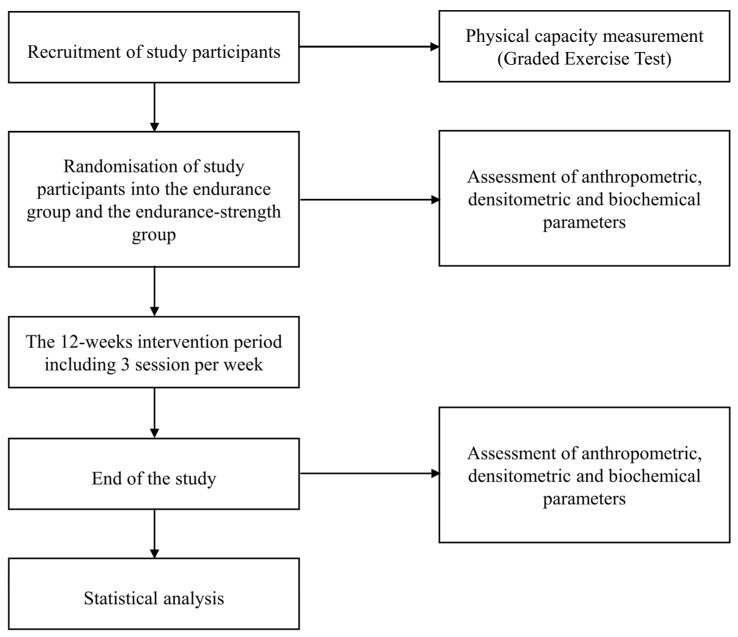
Scheme of the study.

**Figure 2:**
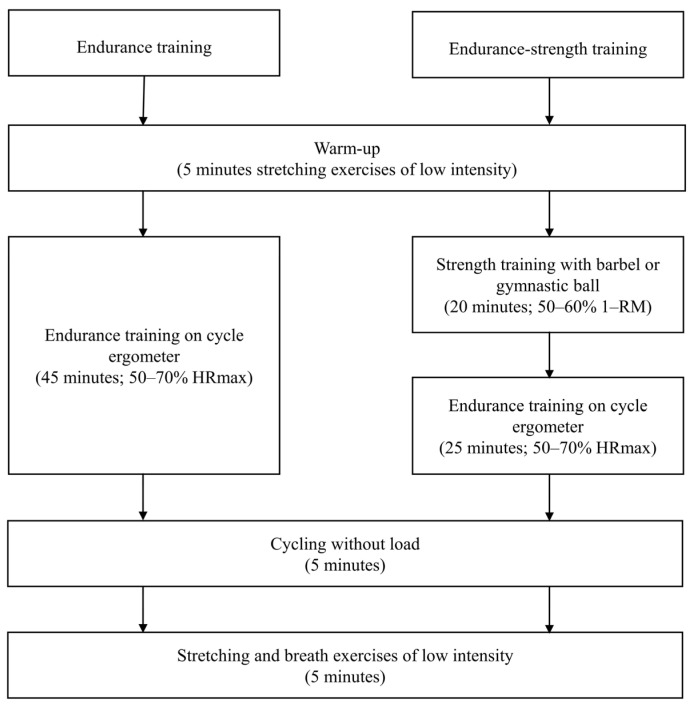
Scheme of a training session.

The scheme of the study is depicted in Figure 1. Participants were randomly assigned to one of two groups in this 12-week study: endurance or endurance-strength (allocation ratio 1:1). Both training programs varied in the nature of the effort but had a similar volume. In both groups, the intervention consisted of 36 training sessions, and the participants had to complete at least 80% of them to be included in the analysis. The endurance group participated in the exercise training sessions three times weekly, which were approximately 60 min long and included 5 min of warm-up, followed by 45 min of endurance exercises, 5 min of cycling without load, and 5 min of stretching and breath exercises. Participants exercised at approximately 50–75% of their maximum heart rate (HR max). The endurance-strength group (also referred to as the combined group) exercised three times weekly, with sessions lasting approximately 60 min and including 5 min of warm-up, followed by 20 min of strength exercises, 25 min of endurance exercises, 5 min of cycling without load, and 5 stretching minutes and breath exercises. The strength exercises involved upper limb exercises performed with a barbell, spine-stabilizing exercises, deep muscle-forming exercises, and balance-adjusting exercises with a gymnastic ball and lower limb exercises with a barbell at 50–60% of their one-repetition maximum. The strength exercises were performed according to the National Strength and Conditioning Association guideline [28] and were repeated in series, with the number of repetitions dependent on the subjects’ muscle strength and systematically increased during the study. The goal number of repetitions was 16 per set in barbell curls and 30 per set in barbell squats. Between the series, a few seconds were taken for breaks. The scheme of a single training session is presented in Figure 2. Here, the 12-week intervention period was applied because we believe that, to observe any effect of training on metabolic, biochemical, and densitometric parameters, at least three months of intervention are needed. Moreover, the intensity of the training sessions in both groups was individually selected for each woman and did not change during the intervention period to obtain a more sustainable effect. All training sessions were supervised by exercise trainers and were performed afternoon. Participants were advised to maintain their usual physical activity outside of exercise sessions. They were also asked not to change dietary habits. No deviations from the intervention were noted. Our previous pilot study also assessed the effect of 12-week endurance and endurance-strength training programs on densitometric parameters (data not published) and physical capacity [29]. However, the study included a small number of abdominally obese women aged 28–62. Additionally, due to the negative effect of training on bone health observed in the pilot study, we decided to modify the training programs and included cycling with a load. Moreover, we decided to narrow the study population’s age range to obtain a more homogeneous group. Moreover, it should be highlighted that the subjects who previously participated in our pilot study were not included in the present study.

### 2.4. Outcomes

The main outcome of the trial was the effect of endurance and endurance-strength training on endothelial function markers (asymmetric dimethylarginine, endothelial nitric oxide synthase, homocysteine, plasminogen activator inhibitor-1, soluble vascular cell adhesion molecule-1, and vascular endothelial growth factor levels) [26]. Here, the effect of endurance and endurance-strength training on secondary outcomes was assessed including differences between groups in the BMD and BMC changes in the whole body, at the lumbar spine, and at the femoral neck; physical capacity; and blood pressure (BP) measured before the graded exercise test (GXT) and at the ventilatory threshold. All measurements, except for GXT, were performed at the Poznan University of Medical Sciences. GXT was performed at the Poznan University of Physical Education.

### 2.5. Anthropometric Parameters

Before the study intervention, several anthropometric parameters, including body weight, height, and waist circumference, were collected. Moreover, BMI was calculated. During the measurements, the participants wore no shoes and minimal clothes. Moreover, an assessment of body composition was performed during the recruitment process to check if subjects fulfilled the inclusion criteria (body fat ≥32%). Body composition was assessed using the bioimpedance method with the InBody 370 analyzer (InBody Co. Ltd., Seoul, Korea).

### 2.6. Densitometry Parameters

BMD and BMC of the total body (BMD and BMC from all parts of the body), lumbar spine (L1-L4), and femoral neck were measured using the dual-energy X-ray absorptiometry method (Hologic Discovery, DXA system, Mississauga, ON, Canada) [26]. The measurements were performed according to the International Society for Clinical Densitometry guideline [30]. Calibration of the analyzer was performed before each scan. All measurements were made by the same certified and blinded assessor. The measurements were performed in the morning. During the assessment, subjects wore only their underwear and were barefoot. The duration of a single measurement was approximately 15 min.

### 2.7. Graded Exercise Test

To determine the study participants’ physical capacity, GXT was performed before and after the intervention period on an electronically braked cycle ergometer (Kettler DX1 Pro, Ense-Parsit, Germany). The test started at a work rate of 25 W and increased incrementally by 25 W every two minutes to the moment when the participant could not maintain the required pedal cadence. During the GXT, expelled gases and minute ventilation were monitored using the Oxycon Mobile system (Viasys Healthcare, Hoechberg, Germany) to determine oxygen intake (VO_2_) and carbon dioxide output (VCO_2_), VO_2_peak, HRpeak, time to exhaustion (TTE), and maximal work rate (WRmax). The ventilatory threshold was assessed using the V-slope and the ventilatory equivalent methods. Moreover, the work rate at the ventilatory threshold (WR_VT_), heart rate at the ventilatory threshold (HR_VT_), and time to the ventilatory threshold (T_VT_) were determined.

### 2.8. Blood Pressure

BP was measured before and during the GXT (systolic blood pressure at the ventilatory threshold (SBP_VT_) and diastolic blood pressure at the ventilatory threshold (DBP_VT_)). BP measured before the GXT was assessed according to guidelines of the European Society of Hypertension [31].

### 2.9. Randomization and Blinding

Randomization was conducted by an independent researcher using Random Allocation Software (Isfahan, Iran). Blocked randomization stratified according to age, body weight, BMI, and waist circumference was applied, and a computer-generated randomization list was generated. The allocation sequence was concealed until participants were enrolled and assigned to the interventions. Participants and careers and subjects delivering the intervention were not blind due to the nature of the intervention. However, neither the outcome assessors nor the data collectors—or the statistician and study team members who prepared the database—were aware of the group assignments.

### 2.10. Minimum Sample Size

The G*Power 3.1.9.7 software (University of Kiel, Kiel, Germany) was used to calculate the minimum sample size. The calculation was performed based on differences in BMD previously reported by Villareal et al. [23]. The sample size required to obtain a power of 80% (α = 0.05, β = 0.2) was 18 subjects per group. Assuming that 20% of subjects may withdraw from the trial, at least 22 individuals should be recruited for each group. Considering our shorter intervention period compared with the study conducted by Villareal et al. [23] (three vs. six months), we decided to include double the number of subjects in our study.

### 2.11. Statistical Analysis

Statistical analyses were performed using STATISTICA software (TIBCO Software Inc., Palo Alto, CA, USA). The data are presented as medians (interquartile ranges (Q1–Q3)) and means (SD) with the 95% confidence interval of means (95% CI). The Shapiro–Wilk test was used to assess the normality of variables and differences among the groups were tested using the Mann–Whitney U test, as most of the data were not normally distributed. Differences between the pre-intervention and post-intervention values were assessed with the Wilcoxon test. *p* < 0.05 was considered statistically significant.

## 3. Results

### 3.1. Participants Flow and Baseline Characteristic

**Figure 3:**
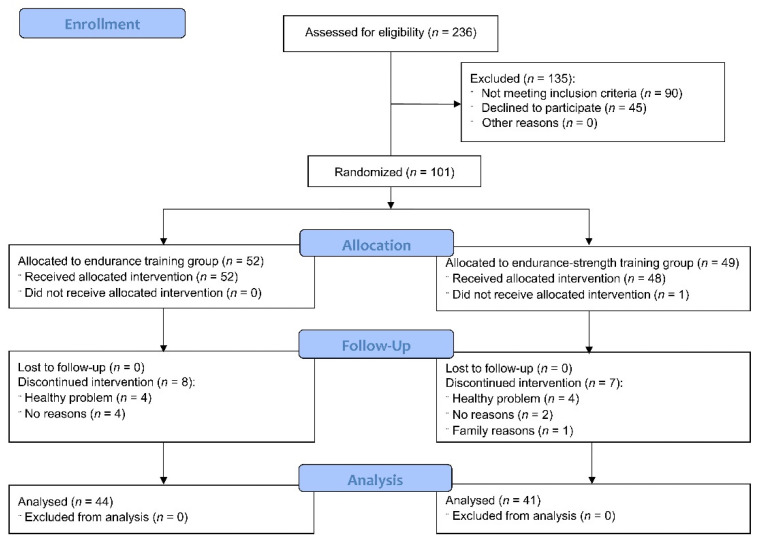
CONSORT 2010 flow diagram [32]. The figure was previously published in the *Journal of Clinical Medicine*, which publishes articles under an open access Creative Common CC BY license.

Subjects were recruited to the study between January and August 2016. Due to organizational possibilities, the intervention period was divided into two sessions: the first started in April 2016 and finished in June 2016, and the second was performed between September and November 2016. Forty-eight women took part in the spring session, and fifty-three subjects participated in the autumn session. Figure 3 presents the participant flow. In total, 236 subjects were assessed for eligibility. Out of them, 135 subjects were excluded (90 women did not meet the inclusion criteria, and 45 women declined to participate). Eventually, 101 subjects were randomly divided into the endurance training group (*n* = 52) and the endurance-strength training group (*n* = 49). Only one subject from the endurance-strength group did not receive the allocated intervention. Moreover, 15 subjects (eight from the endurance group and seven from the combined group) discontinued the intervention (eight were due to health problems, six did not provide reasons, and one declined due to personal reasons). A total of 85 postmenopausal women (84%; the endurance group: *n* = 44, the endurance-strength training group: *n* = 41) completed the study and were included in the final analysis. The mean adherence was 91%. No severe side effects were reported. Six women had joint or muscle pain, two subjects reported high blood pressure, and one participant had swelling. There were no significant differences among the groups at baseline, as shown in Table 1 and Table 2.

### 3.2. The Effect of Endurance and Endurance-Strength Training on Densitometric Parameters

The effects of both types of training on BMC and BMD are shown in Table 3. BMC and BMD did not change at the femoral neck, at the lumbar spine, and in the whole body in the endurance training group. Similarly, endurance-strength training did not result in BMC changes at any of the measurement regions. In contrast to the femoral neck and lumbar spine, total body BMD increased significantly after the intervention.

### 3.3. The Effect of Endurance and Endurance-Strength Training on Physical Capacity

The effects of endurance and endurance-strength training on physical capacity are presented in Table 4. Both training types led to a significant increase in all parameters assessed during the GXT. Both types of training did not affect SBP, while a decrease in DBP and an increase in SBP_VT_ were observed. Moreover, a significant reduction in DBP_VT_ was observed for combined but not endurance training.

### 3.4. Comparison of the Effect of Endurance and Endurance-Strength Training on Densitometric Parameters and Physical Capacity

Table 5 and Table 6 show comparisons of the impact of endurance and endurance-strength training on densitometric parameters and physical capacity. There were no differences between the effect of these types of training on BMD (all measured parameters) and BMC at the femoral neck and in whole body. However, the endurance training was more favorable than the endurance-strength training program for maintaining BMC at the lumbar spine (mean (95% CI): 0.84 (−0.50 to 2.16) vs. −0.32 (−1.68 to 0.35), *p* = 0.04). Furthermore, no differences between the effects of endurance and endurance-strength training on physical capacity, BP measured before GXT, and BP_VT_ were documented.

## 4. Discussion

The major finding of the present study was that endurance training was more effective in maintaining BMC at the lumbar spine. However, both types of training did not differ in effect on BMD and positively affected physical capacity. This is one of the first studies that compared the effect of endurance and endurance-strength training on densitometric parameters in abdominally obese women.

Previous studies that assessed the effect of endurance, strength, and combined training on densitometric parameters provided equivocal results. Liang et al. [19] demonstrated that 12-month high-impact step endurance exercises performed three times a week per 60 min under supervision resulted in a significant increase in the heel BMD in untrained young women who complied with the exercise regimen. However, a moderate intensity strength training intervention of similar duration and frequency did not affect the areal BMD. By contrast, Beavers et al. [22] suggested that performing strength training three days per week rather than endurance training (treadmill walking) underwent four days per week during caloric restriction may attenuate loss of hip and femoral neck BMD in overweight and obese older adults. Heinonen et al. [33] assessed the effect of 18 months of high-impact exercises consisting of either an endurance (jump training) or a step program performed three times a week in sedentary women aged 35–45 years and noted that BMD at the lumbar spine, femoral neck, distal femur, patella, proximal tibia, and calcaneus was significantly greater in the training group than in the control group. Furthermore, Friedlander et al. [34] performed studies for two years using three sets of one-hour endurance and weight training a week in young women and found significant differences in BMD between groups for spinal trabecular, femoral neck, femoral trochanteric and calcaneal measurements. Campanha-Versiani et al. [35] also found that, in patients with obesity who underwent gastric bypass surgery, the endurance training performed on a treadmill and weight-bearing program (both performed twice weekly for 36 weeks) compared with the control group attenuated lumbar spine and right hip BMD loss but did not affect BMC. Moreover, Lim et al. [36] compared the effect of an eight-week combined training that included stair climbing, cycling, and resistance exercises between the obese and non-obese groups and reported that the total hip BMC and BMD slightly increased after the intervention period only in obese subjects. However, in both groups, no changes were observed for BMC and BMD at the lumbar spine, femoral neck, and trochanter. In general, it seems that high-intensity and speed endurance training can limit the reduction in BMD. At the same time, strength exercises have a site-specific effect and can increase BMD only in the stimulated body regions. However, to make endurance-strength programs effective, the training must contain an adequate proportion of endurance and resistance exercises [37]. Our study showed no differences between pre- and post-intervention values of BMC and BMD at the femoral neck, lumbar spine, and total body in the endurance training group, while in the endurance-strength training group, total body BMD increased significantly during the intervention. These findings are consistent with a previous systematic review that showed that the combined exercises have a significant effect on BMD in postmenopausal women [38]. However, we found that there were significant differences between the effect of both training programs on BMC at the lumbar spine (L1–L4).

Until now, only a few studies have compared the effect of endurance and endurance-strength training on bone health. In a group of dieting older adults with obesity, Villareal et al. [23] reported that BMD at the total hip did not change significantly after 26 weeks of strength training but decreased in the endurance and the endurance-strength groups, with a higher reduction observed in the endurance group. However, the combined training lasted much longer than endurance or strength training alone. The sessions of combined training were 75 to 90 min long, while in the endurance and strength group, the duration of training was approximately 60 min. Moreover, the BMD of the whole body and at the lumbar spine did not change significantly in any of the groups. Moreover, Campos et al. [21] assessed the effect of similar duration aerobic and combined exercises and found that both training programs did not affect BMD but that mixed training significantly improved BMC in post-pubertal obese adolescents. Contrary, Stensvold et al. [24] reported no effect of 12-week endurance, strength, or endurance-strength training (all of a similar duration) on BMC (with no difference between them) in subjects with metabolic syndrome. The presented findings are contrast with our results as we found that both types of training did not differ in effect on BMD; however, endurance training was more effective in maintaining BMC at the lumbar spine.

Several factors can explain the differences between our results and previous findings. First, the effect of endurance and endurance-strength training on densitometric parameters might vary in different age groups. Indeed, exercise induces an increase in bone mass in younger subjects. However, this effect in adults and elderly subjects remains questionable [39]. Second, the effect of exercises on bone health might differ in men and women. Indeed, Almstedt et al. [40] observed that men had significantly greater increases in BMD at the lateral spine and femoral neck after 24 weeks of resistance training. Furthermore, in women, the effect of endurance and endurance-strength training on bone health might differ in before and after menopause. In a recent meta-analysis, Martyn-St. James et al. [14] found that high-intensity resistance training significantly increased BMD in postmenopausal women compared with the non-exercise control group. However, the same authors in another meta-analysis that included studies conducted in premenopausal women compared with controls demonstrated that high-intensity progressive resistance training increased BMD at the lumbar spine but not the femoral neck [15]. Furthermore, in these studies, factors such as initial bone status, subject selection, sample sizes, and concomitant hormone therapy were identified as factors confounding interpretation of obtained results [14,15]. It has been suggested that higher intensity and longer study periods may be important factors to consider for increasing BMD and BMC [33]. Indeed, Tsuzuku et al. [16] reported that high-intensity resistance training improved BMD for the total body and at trochanter regions compared with the low-intensity and control groups. A previous report also suggested that bone remodeling requires at least four to six months [41]. However, a beneficial effect of exercises on bone health was reported in studies with longer [19,33,34] and shorter [36] intervention periods compared with our study. Moreover, Marcus [42] suggested that the volume of exercise is a major factor in enhancing skeletal BMD. High-frequency and low-impact loading (e.g., walking, standing, and stepping) can effectively induce an osteogenic response in lower limbs. In our study, both training programs had a similar volume and varied only in the nature of the effort, which may partly explain the lack of significant differences in the effect of both types of training on BMD. Different types of endurance training may also have a different effect on bone health. It seems that cycling, swimming, and walking do not positively affect bone health [37,43,44]. However, high-intensity and speed endurance training (e.g., jogging, climbing, and stepping) may prevent BMD reduction [37]. The magnitude in BMD and BMC changes found in the selected studies may also be explained by differences in the muscle strength of the study subjects, demographic profiles, and methodological differences. Additionally, it should be noted that, in some previous studies, the exercise program was combined with weight loss therapy. However, it is well known that caloric restriction might affect bone health [2]. Inconsistent results might also depend on the vitamin D and calcium intake of study participants [45]. Furthermore, the place of the measurement can also affect obtained results [33,46]. Notwithstanding, it should be noted that BMD should be measured at the femoral neck and/or lumbar spine. Examinations performed at other sites of the body should not be used to diagnose osteoporosis [30,47].

It is well known that obesity has an unbeneficial effect on physical capacity [48] and that regular training favorably affects these parameters [49]. Indeed, our results also showed that both training programs were similarly effective in the improvement of physical capacity. Similar results were obtained in our previous pilot study. In both groups, we noted significant increases in VO_2_peak, TTE, WRmax, and WR_VT_, with no significant differences observed between groups for the investigated parameters [29]. However, several previous studies noted that the type of training might have a significant impact on the effect of exercise on physical capacity [49,50,51]. Schjerve et al. [50] found that the physical capacity parameters increased significantly in the high-intensity endurance training group when compared with the strength group. Hendrickson et al. [51] reported that VO_2_peak increased in the endurance-strength group and the endurance group but not in the strength group or the control group. In another study, a significant increase in VO_2_peak and VO_2_peak/kg were found both in the endurance and the combined training group but not in the strength group. However, the VO_2_peak/lean body mass index increased in only the combined training group [49]. By contrast, Jorge et al. [52] reported that endurance training but not resistance and combined training increased the VO_2_peak. The lack of difference in the effect of endurance and endurance-strength training on physical capacity observed in our study can be partially explained by the similar volume and duration of the single training session for both types of programs as well as the similar impact of both types of training on anthropometric parameters [32].

Here, we also noticed that both types of exercises significantly decreased DBP measured before GXT and increased SBP_VT_ but did not affect SBP measured before GXT. Moreover, the combined training significantly reduced DBP_VT_ but this effect was not observed in the endurance group. However, we did not find any differences between groups. In our pilot study [29], we demonstrated a decrease in resting SBP, resting, and exercise DBP in the endurance group and the endurance-strength training group with no difference between training programs. Similarly, Kargarfard et al. [53] reported improvement in resting and exercise SBP and resting DBP after two months of an aerobic training program in patients after myocardial infarction. The beneficial effect of both endurance and endurance-strength training on BP was also observed in a recent meta-analysis [54]. Several mechanisms explaining the reduction in BP after the intervention have been proposed. It is well known that exercises decrease catecholamine levels and resting HR [55]. The decrease in renin and norepinephrine levels might also affect the antihypertension effect of exercise [56]. Other factors such as decreased sympathetic nervous system activity and increased sensitivity of the baroreceptor reflex after exercises might also have a beneficial effect on BP reduction [53].

Several study limitations warrant acknowledgment. First, implementing the intervention into home conditions might be not effective due to a lack of supervision. The absence of direct bone metabolism measurements could also be regarded as a limitation. Moreover, we did not analyze several factors that could affect bone health, such as prior or concurrent physical activity, calcium consumption, and serum 25-hydroxyvitamin D concentrations. The lack of a strength group could also be taken as a limitation. Finally, our findings apply only to abdominally obese postmenopausal women aged 50–60, so cannot be generalized to the total population. Another limitation is the short study duration, as bone remodeling is a slow process, requiring a minimum of four to six months [41]. Furthermore, we did not assess daily physical activity outside of exercise sessions. Therefore, we were not able to check if participants maintain their usual physical activity level. However, the strengths of this study include a well-characterized study population and very strict inclusion and exclusion criteria. Moreover, the randomized design and the high rate of adherence to the trial interventions should be noted.

## 5. Conclusions

In conclusion, our study showed that endurance training was more effective in maintaining BMC at the lumbar spine in abdominally obese postmenopausal women. However, both types of training improved physical capacity and did not differ in effect on BMD. Further studies with a long-term follow-up should be considered to confirm these findings.

## Figures and Tables

**Table 1:** Baseline characteristic of the study population (*n* = 101).

		Endurance (*n* = 52)		Endurance-Strength (*n* = 49)		*p*
		Median (Q1–Q3)	Mean ± SD (95% CI)	Median (Q1–Q3)	Mean ± SD (95% CI)	
Age [years]		55 (50–60)	55 ± 7 (53 to 57)	54 (50–60)	55 ± 7 (53 to 58)	0.84
Body weight [kg]		93.4 (84.9–104.9)	96.0 ± 15.1 (91.7 to 100.2)	91.0 (82.4–101.8)	93.2 ± 13.9 (89.2 to 97.2)	0.41
BMI [kg/m^2^]		35.64 (32.07–38.00)	35.87 ± 4.43 (34.63 to 37.10)	35.42 (31.79–39.10)	35.98 ± 5.10 (34.52 to 37.45)	0.86
Waist circumference [cm]		109 (103–114)	110 ± 10 (107 to 113)	108 (103–117)	110 ± 10 (107 to 113)	1.00
Femoral neck	BMC [g]	4.29 (4.07–4.67)	4.38 ± 0.67 (4.19 to 4.57)	4.56 (3.86–4.84)	4.45 ± 0.77 (4.23 to 4.67)	0.61
	BMD [g/cm^2^]	0.866 (0.805–0.945)	0.870 ± 0.120 (0.836 to 0.903)	0.906 (0.792–0.995)	0.901 ± 0.135 (0.862 to 0.940)	0.26
Lumbar spine (L1–L4)	BMC [g]	59.20 (52.73–66.24)	60.20 ± 11.02 (57.13 to 63.26)	65.14 (52.60–68.11)	63.12 ± 11.38 (59.84 to 66.38)	0.18
	BMD [g/cm^2^]	1.028 (0.936–1.146)	1.035 ± 0.133 (0.998 to 1.072)	1.072 (0.972–1.160)	1.080 ± 0.126 (1.044 to 1.116)	0.08
Total	BMC [g]	2369.94 (2240.06–2565.25)	2417.98 ± 349.84 (2320.59 to 2515.38)	2447.10 (2247.34–2714.55)	2466.74 ± 344.62 (2367.75 to 2565.72)	0.37
	BMD [g/cm^2^]	1.179 (1.115–1.236)	1.173 ± 0.098 (1.145 to 1.200)	1.178 (1.113–1.293)	1.201 ± 0.115 (1.168 to 1.234)	0.30

BMC, bone mineral content; BMD, bone mineral density; BMI, body mass index; Q1–Q3, interquartile range; and 95% CI, 95% confidence interval of means.

**Table 2:** Baseline physical capacity of the study population (*n* = 101).

		Endurance (*n* = 52)		Endurance-Strength (*n* = 49)		*p*
		Median (Q1–Q3)	Mean ± SD (95% CI)	Median (Q1–Q3)	Mean ± SD (95% CI)	
VT values	WR_VT_ [W]	75 (50–100)	76 ± 21 (71 to 82)	75 (50–87)	73 ± 20 (67 to 80)	0.53
	HR_VT_ [bpm]	121 (112–132)	122 ± 13 (118 to 126)	127 (115–133)	124 ± 15 (119 to 128)	0.31
	T_VT_ [min]	7 (5–8)	7 ± 2 (6 to 7)	6 (5–7)	7 ± 2 (6 to 7)	0.31
Peak values	VO_2_peak [ml/(kg × min)]	15.7 (14–17)	16.0 ± 3.1 (15.2 to 16.9)	15.7 (1.9–17.6)	15.9 ± 3.0 (15.0 to 16.7)	0.99
	WRmax [W]	125 (100–125)	117 ± 29 (109 to 125)	100 (75–125)	109 ± 30 (100 to 117)	0.24
	HRpeak [bpm]	140 (132–150)	142 ± 17 (138 to 147)	146 (126–158)	143 ± 20 (137 to 149)	0.40
	TTE [min]	10 (8–11)	10 ± 2 (9 to 10)	9 (7–10)	9 ± 2 (8 to 10)	0.23
BP	SBP ^a^ [mmHg]	130 (125–145)	134 ± 15 (130 to 138)	135 (130–140)	135 ± 12 (131 to 138)	0.68
	DBP ^a^ [mmHg]	80 (80–90)	84 ± 9 (81 to 86)	85 (80–90)	83 ± 9 (80 to 85)	0.94
	SBP_VT_ [mmHg]	170 (150–190)	174 ± 21 (168 to 179)	170 (160–180)	171 ± 20 (166 to 177)	0.62
	DBP_VT_ [mmHg]	90 (80–95)	86 ± 13 (83 to 90)	90 (80–95)	87 ± 11 (84 to 90)	0.89

^a^ Blood pressure measured before GXT; BP, blood pressure, DBP, diastolic blood pressure; DBP_VT_, diastolic blood pressure at the ventilatory threshold; HRpeak, peak heart rate; HR_VT_, ventilatory threshold heart rate; Q1–Q3, interquartile range; SBP, systolic blood pressure; SBP_VT_, systolic blood pressure at the ventilatory threshold; TTE, time to exhaustion; T_VT_, time to the ventilatory threshold; VO_2_peak, peak oxygen intake; VT, ventilatory threshold; WRmax, maximal work rate; WR_VT_, ventilatory threshold work rate; and 95% CI, 95% confidence interval of means.

**Table 3:** The effect of endurance (*n* = 44) and endurance-strength training (*n* = 41) on densitometric parameters.

		Endurance *(n* = 44)				*p*	Endurance-Strength (*n* = 41)				*p*
		Pre-Intervention		Post-Intervention			Pre-Intervention		Post-Intervention		
		Median (Q1–Q3)	Mean ± SD (95% CI)	Median (Q1–Q3)	Mean ± SD (95% CI)		Median (Q1–Q3)	Mean ± SD (95% CI)	Median (Q1–Q3)	Mean ± SD (95% CI)	
Femoral neck	BMC (g)	4.29 (4.08–4.67)	4.36 ± 0.65 (4.16 to 4.55)	4.31 (4.08–4.73)	4.35 ± 0.6 (4.14 to 4.55)	0.77	4.56 (3.86–4.90)	4.47 ± 0.79 (4.22 to 4.72)	4.49 (3.87–4.94)	4.48 ± 0.81 (4.23 to 4.74)	0.92
	BMD (g/cm^2^)	0.860 (0.802–0.937)	0.863 ± 0.118) (0.827 to 0.899)	0.846 (0.792–0.933)	0.861 ± 0.124 (0.823 to 0.899)	0.96	0.904 (0.805–0.995)	0.901 ± 0.134 (0.859 to 0.943)	0.886 (0.790–1.004)	0.898 ± 0.135 (0.855 to 0.940)	0.44
Lumbar spine(L1–L4)	BMC (g)	59.83 (52.73–65.71)	60.04 ± 10.56 (56.83 to 63.25)	60.20 (54.24–64.84)	60.87 ± 10.69 (57.62 to 64.11)	0.08	65.14 (53.56–68.11)	63.48 ± 11.83 (59.75 to 67.21)	62.81 (53.32–69.07)	62.81 ±11.35 (59.23 to 66.40)	0.31
	BMD (g/cm^2^)	1.029 (0.938–1.150)	1.041 ± 0.137 (0.999 to 1.082)	1.029 (0.931–1.154)	1.044 ± 0.133 (1.004 to 1.085)	0.35	1.072 (0.963–1.160)	1.079 ± 0.132 (1.037 to 1.120)	1.083 (0.956–1.151)	1.071 ± 0.127 (1.031 to 1.112)	0.17
Total	BMC (g)	2369.94 (2252.26–2552.05)	2425.88 ± 360.03 (2316.42 to 2535.34)	2358.48 (2255.76–2570.16)	2431.95 ± 365.52 (2320.82 to 2542.08)	0.53	2436.41 (2247.34–2681.05)	2457.36 ± 347.60 (2347.64 to 2567.07)	2450.18 (2221.46–2716.82)	2466.69 ± 348.34 (2356.74 to 2576.64)	0.25
	BMD (g/cm^2^)	1.179 (1.104–1.222)	1.169 ± 0.100 (1.138 to 1.199)	1.176 (1.255–1.242)	1.174 ± 0.103 (1.143 to 1.206)	0.16	1.169 (1.113–1.274)	1.197 ±0.118 (1.160 to 1.234)	1.191 (1.115–1.278)	1.205 ±0.116 (1.168 to 1.241)	0.02

BMC, bone mineral content; BMD, bone mineral density; BMI, body mass index; Q1–Q3, interquartile range; and 95% CI, 95% confidence interval of means.

**Table 4:** The effect of endurance (*n* = 44) and endurance-strength training (*n* = 41) on physical capacity.

		Endurance *(n* = 44)				*p*	Endurance-Strength (*n* = 41)				*p*
		Pre-Intervention		Post-Intervention			Pre-Intervention		Post-Intervention		
		Median (Q1–Q3)	Mean ± SD (95% CI)	Median (Q1–Q3)	Mean ± SD (95% CI)		Median (Q1–Q3)	Mean ± SD (95% CI)	Median (Q1–Q3)	Mean ± SD (95% CI)	
VT values	WR_VT_ [W]	75 (50–100)	76 ± 20 (70 to 82)	100 (100–125)	105 ± 24 (97 to 112)	<0.001	75 (50–100)	75 ± 21 (68 to 82)	100 (100–100)	100 ± 21 (93 to 107)	<0.001
	HR_VT_ [bpm]	124 (110–132)	122 ± 13 (118 to 127)	129 (120–137)	127 ± 13 (123 to 131)	<0.001	127 (118–132)	124 ± 15 (119 to 129)	131 (123–139)	130 ± 14 (125 to 134)	<0.001
	T_VT_ [min]	7 (5–8)	7 ± 2 (6 to 7)	9 (8–10)	9 ± 2 (8 to 10)	<0.001	6 (5–8)	7 ± 2 (6 to 7)	9 (8–9)	9 ± 2 (8 to 9)	<0.001
Peak values	VO_2_peak [ml/(kg × min)]	15.9 (14.2–17.5)	16.2 ± 3.0 (15.3 to 17.1)	19.1 (17.6–22.0)	19.7 ± 3.4 (18.7 to 20.8)	<0.001	15.9 (14.1–17.8)	16.2 ± 2.9 (15.3 to 17.1)	19.4 (16.8–22.1)	19.7 ± 3.4 (18.6 to 20.8)	<0.001
	WRmax [W]	125 (100–125)	119 ± 28 (110 to 127)	150 (125–175)	148 ± 34 (138 to 159)	<0.001	125 (100–125)	112 ± 31 (102 to 121)	150 (125–175)	148 ± 25 (140 to 156)	<0.001
	HRpeak [bpm]	143 (134–152)	143 ± 16 (138 to 148)	147 (139–157)	148 ± 16 (143 to 153)	0.02	148 (134–158)	145 ± 19 (139 to 151)	155 (141–164)	152 ± 17 (147 to 157)	<0.001
	TTE [min]	10 (8–11)	10 ± 2 (9 to 11)	12 (10–13)	12 ± 3 (11 to 13)	<0.001	10 (7–10)	9 ± 2 (9 to 10)	12 (11–14)	12 ± 2 (12 to 13)	<0.001
BP	SBP ^a^ [mmHg]	130 (125–145)	135 ± 15 (130 to 140)	130 (120–140)	131 ± 14 (127 to 135)	0.26	135 (130–140)	135 ± 11 (132 to 139)	130 (120–140)	129 ± 16 (124 to 134)	0.07
	DBP ^a^ [mmHg]	80 (80–90)	84 ± 9 (81 to 87)	80 (75–85)	79 ± 7 (76 to 81)	0.007	85 (80–90)	84 ± 8 (81 to 86)	80 (70–80)	76 ± 7 (74 to 78)	<0.001
	SBP_VT_ [mmHg]	180 (160–190)	175 ± 20 (168 to 181)	190 (175–205)	188 ± 21 (182 to 195)	<0.002	170 (160–180)	172 ± 19 (166 to 178)	180 (170–200)	183 ± 22 (176 to 189)	0.006
	DBP_VT_ [mmHg]	90 (80–95)	86 ± 14 (82 to 90)	82 (80–90)	84 ± 10 (81 to 87)	0.48	90 (85–95)	89 ± 10) (85 to 92)	80 (75–90)	82 ± 12 (78 to 86)	0.006

^a^ Blood pressure measured before GXT; BP, blood pressure, DBP, diastolic blood pressure; DBP_VT_, diastolic blood pressure at the ventilatory threshold; HRpeak, peak heart rate; HR_VT_, ventilatory threshold heart rate; Q1–Q3, interquartile range; SBP, systolic blood pressure; SBP_VT_, systolic blood pressure at the ventilatory threshold; TTE, time to exhaustion; TVT, time to the ventilatory threshold; VO_2_peak, peak oxygen intake; VT, ventilatory threshold; WRmax, maximal work rate; WR_VT_, ventilatory threshold work rate; and 95% CI, 95% confidence interval of means.

**Table 5:** Comparison of the effect of endurance (*n* = 44) and endurance-strength training (*n* = 41) on densitometric parameters.

		Endurance (*n* = 44)		Endurance-Strength *(n* = 41)		*p*
		Median (Q1–Q3)	Mean (SD) (95% CI)	Median (Q1–Q3)	Mean (SD) (95% CI)	
Femoral neck	Δ BMC [g]	−0.01 (−0.16–0.10)	−0.01 ± 0.24 (−0.08 to 0.06)	−0.03 (−0.13–0.13)	0.01 ± 0.21 (−0.05 to 0.08)	0.80
	Δ BMD [g/cm^2^]	0.002 (−0.014–0.012)	−0.002 ± 0.022 (−0.009 to 0.005)	0.002 (−0.023–0.012)	−0.003 ± 0.032 (−0.014 to 0.007)	0.57
Lumbar spine (L1–L4)	Δ BMC [g]	0.84 (−0.73–2.31)	0.83 ± 4.38 (−0.50 to 2.16)	−0.32 (−1.89–0.99)	−0.67 ± 3.23 (−1.68 to 0.35)	0.04
	Δ BMD [g/cm^2^]	0.006 (−0.013–0.025)	0.004 ± 0.039 (−0.008 to 0.016)	−0.008 (−0.021–0.011)	−0.007 ± 0.030 (−0.017 to 0.002)	0.10
Total	Δ BMC [g]	6.35 (−26.75–36.28)	6.07 ± 49.70 (−9.04 to 21.19)	10.22 (−27.91–38.86)	9.33 ± 49.95 (−6.44 to 25.09)	0.71
	Δ BMD [g/cm^2^]	0.003 (−0.009–0.014)	0.006 ± 0.022 (−0.001 to 0.012)	0.008 (0.003–0.022)	0.008 ± 0.020 (0.001 to 0.014)	0.42

BMC, bone mineral content; BMD, bone mineral density; BMI, body mass index; Q1–Q3, interquartile range; and 95% CI, 95% confidence interval of means.

**Table 6:** Comparison of the effect of endurance (*n* = 44) and endurance-strength training (*n* = 41) on physical capacity.

		Endurance (*n* = 44)		Endurance-Strength (*n* = 41)		*p*
		Median (Q1–Q3)	Mean ± SD (95% CI)	Median (Q1–Q3)	Mean ± SD (95% CI)	
VT values	Δ WR_VT_ [W]	25 (25–50)	30 ± 13 (26 to 35)	25 (25–25)	26 ± 15 (21 to 31)	0.31
	Δ HR_VT_ [bpm]	6 (−1–13)	6 ± 9 (3 to 9)	5 (1–14)	6 ± 10 (3 to 9)	0.82
	Δ T_VT_ [min]	2 (2–3)	2 ± 1 (2 to 3)	2 (1–3)	2 ± 1 (2 to 2)	0.26
Peak values	Δ VO_2_peak [ml/(kg × min)]	3.2 (2.2–5.1)	3.5 ± 2.2 (2.8 to 4.1)	3.7 (2.1–4.5)	3.5 ± 1.9 (2.9 to 4.1)	0.99
	Δ WRmax [W]	25 (25–50)	29 ± 23 (22 to 36)	25 (25–50)	37 ± 19 (31 to 42)	0.15
	Δ HRpeak [bpm]	5 (−4–13)	5 ± 12 (1 to 9)	7 (0–16)	7 ± 11 (3 to 10)	0.39
	Δ TTE [min]	2 (1–3)	2 ± 2 (2 to 3)	3 (2–4)	3 ± 1 (2 to 3)	0.11
BP	Δ SBP ^a^ [mmHg]	0 (−20–10)	−3 ± 16 (−9 to 2)	−5 (−10–5)	−6 ± 17 (−11 to 0)	0.49
	Δ DBP ^a^ [mmHg]	−5 (−10–0)	−5 ± 11 (−8 to−2)	−5 (−15–0)	−8 ± 11 (−11 to −4)	0.34
	Δ SBP_VT_ [mmHg]	25 (25–50)	30 ± 13 (26 to 35)	25 (25–25)	26 ± 15 (21 to 31)	0.31
	Δ DBP_VT_ [mmHg]	6 (−1–13)	6 ± 9 (3 to 9)	5 (1–14)	6 ± 10 (3 to 9)	0.82

^a^ Blood pressure measured before GXT; BP, blood pressure, DBP, diastolic blood pressure; DBP_VT_, diastolic blood pressure at the ventilatory threshold; HRpeak, peak heart rate; HR_VT_, ventilatory threshold heart rate; Q1–Q3, interquartile range; SBP, systolic blood pressure; SBP_VT_, systolic blood pressure at the ventilatory threshold; TTE, time to exhaustion; T_VT_, time to the ventilatory threshold; VO_2_peak, peak oxygen intake; VT, ventilatory threshold; WRmax, maximal work rate; WR_VT_, ventilatory threshold work rate; and 95% CI, 95% confidence interval of means.

## Data Availability

The data presented in this study are available from the corresponding author upon request. The data are not publicly available due to the disagreement of the study participants.

## Supplementary Materials

The following are available online at https://www.mdpi.com/article/10.3390/healthcare9081074/s1, Table S1: CONSORT 2010 checklist of information to include when reporting a randomized trial.
